# Investigating influencing factors on premenstrual syndrome (PMS) among female college students

**DOI:** 10.1186/s12905-023-02752-y

**Published:** 2023-11-10

**Authors:** Su Jeong Yi, Miok Kim, Ina Park

**Affiliations:** 1 Department of Nursing, Dankook University, 119, Dandae-ro, Dongnam-gu, Cheonan-si, Chungcheongnam-do Korea; 2 Center for Sport Science in Chungnam, 370-24, Nambu-ro, Asan-si, Chungcheongnam-do Republic of Korea

**Keywords:** Premenstrual syndrome, Dysmenorrhea, Psychological stress, Depression, Sleep, Eating disorder

## Abstract

**Background:**

Premenstrual syndrome (PMS) affects women’s physical and mental health. Depression, stress, sleep disturbance, and eating attitude problems have been known to influence PMS. Furthermore, restrictions of daily life due to the COVID-19 pandemic have led to changes in sleep patterns and eating attitudes. Thus, it is necessary to closely examine how these factors affect PMS. This study aimed to examine the levels of PMS, stress, depression, sleep disturbance, and eating attitude problems among female college students who experience dysmenorrhea and determine the factors associated with PMS.

**Methods:**

A cross-sectional online survey design was conducted using a convenience sample of 143 female college students in C City, South Korea. Data were collected from September 1 to 19, 2021 in South Korea using an online self-administered survey. Differences in participants’ level of PMS according to physical health variables (e.g., smoking, water intake, menstrual pain intensity) and psychological issues (i.e., stress, depression, sleep disturbances, and eating attitude problems) were assessed with independent sample t-tests and one-way ANOVAs. Correlational analyses between these variables were also conducted. Additionally, multiple regression was performed to identify the factors influencing PMS.

**Results:**

PMS severity was between normal (27.3%) and premenstrual dysphoric disorder (PMDD) (72.7%). PMS was associated positively with depression (r = .284, *p* = 001), stress (r = .274, *p* = .001), sleep disturbance (r = .440, *p* < .001), and eating attitude problems (r = .266, *p* = .001). Additionally, menstrual pain intensity (*β* = 0.204), sleep disturbances (*β* = 0.375), and eating attitude problems (*β* = 0.202) were found to influence PMS. The regression model was significant (F = 16.553, *p* < .001) with an explanatory power of 24.7%.

**Conclusions:**

Considering the influencing factors of PMS identified in this study, interventions for participants experiencing PMS should be made. We propose that further study should be conducted to examine whether the severity of PMS changes according to menstrual pain, the pattern and degree of its change, and the paths through which sleep quality and eating attitude problems affect PMS.

## Introduction

Premenstrual syndrome (PMS) is a combination of more than 200 complex symptoms including physical symptoms, behavioral symptoms, and emotional symptoms [1], which develop approximately 2–10 days before the period and disappear just before or shortly after menstruation begins [2]. Even though the etiology of PMS is unclear, a wide variety of factors seem to come into play, including interactions between biological factors (e.g., hormonal imbalance and neurotransmitter changes) [3] and psychosocial factors (e.g., attitude toward menstruation and stress) [4] as well as routine health behaviors (e.g., exercise, smoking, drinking, sleep duration, diet, and nutrient intake) [1]. PMS is experienced by 99% of Korean women of reproductive age (15 to 49 years), and 13.6 to18.6% of them are diagnosed with premenstrual dysphoric disorder (PMDD) [5], which is included in the depressive disorders chapter of the Diagnostic and Statistical Manual of Mental Disorders, Fifth Edition (DSM-5) [6]. Specifically, women 21–25 years old (college students) have a higher incidence of PMS than high school students and middle-aged women [7]. Although 28.0% of female college students experience severe PMS-induced stress [8] and disturbances in everyday life, they rarely receive professional care [9].

The COVID-19 pandemic has had a major impact on reproductive health, with previous studies indicating changes in menstrual cycles, including regularity, frequency, duration, and PMS symptoms, due to COVID-19-related stress [2, 10]. Stress from COVID-19 can disrupt glucocorticoid hormone levels and affect menstrual cycle regularity [11]. Among such negative emotions, stress can further exacerbate emotional and psychological symptoms associated with PMS. And depression is known to have the highest influence on PMS [4]. Because women at risk of depression tend to have a high likelihood of PMS, it is necessary to assess the risk of depression in women with PMS [12]. PMS should be considered together with psychological and emotional problems experienced by women.

The COVID-19 lockdown has also influenced lifestyle habits, leading to changes in dietary habits, physical activity, sleep patterns, and psychological well-being [13]. Women experiencing PMS are particularly susceptible to sleep disturbances due to hormonal changes associated with the menstrual cycle [14]. They may face difficulties in various aspects of sleep, such as duration, quality, latency, maintenance, and wake-up time, compared to women unaffected by PMS [14]. Additionally, a higher proportion of women with PMS suffer from poor sleep quality compared to those without PMS (75.6% vs. 58.8%) [15], and women with sleep disorders have a 1.7-fold risk of experiencing PMS compared to women without sleep disorders [16], highlighting the close association between PMS and sleep quality.

Eating attitude problems have been linked to PMS [17], and differences in appetite and eating habits are observed between healthy women and those with PMS [18]. Particularly, female college students with PMS are significantly associated with eating attitude problems and depression [19]. Moreover, hospitalizations for eating behavior-related problems, such as anorexia nervosa and other eating disorders, have increased during the COVID-19 pandemic [20, 21]. Thus, understanding the relationship between eating habits and PMS in female college students is crucial.

Previous research [22, 23] various related factors have been investigated focusing on the incidence and severity of PMS. In this study, we examine the levels of PMS, stress, depression, sleep disturbances, and eating attitude problems in female college students who experience dysmenorrhea and determine influencing factors associated with PMS. The findings will contribute to developing efficient nursing intervention programs for preventing and managing PMS.

## Methods

### Study design

This study is a cross-sectional survey research aimed at exploring factors related to PMS among female college students.

### Participants

The inclusion criteria for the study participants are unmarried female college students in their 20s who have experienced menstrual pain, understand the purpose of the research, and can respond to the survey. The exclusion criteria for the study participants are those who have not experienced menstrual pain, have a history of childbirth, or are currently undergoing treatment for mental health issues. They were recruited based on the research findings that the more severe the dysmenorrhea, the more severe the PMS symptoms [24] and that 90.2% of female college students experience weak to strong dysmenorrhea [25]. The minimum sample size of this study estimated using the G*Power 3.1 program was 153 participants for power 0.80, effect size 0.15, and significance level 0.05 with 19 predictors. Considering a dropout rate of 10%, the online questionnaire survey remained open until the number of respondents reached 170, and after removing the respondents who gave uniform answers to all items, data from 143 respondents were finally used for analysis in this study.

### Data collection

At the time of data collection for this research, all university classes had transitioned to online format due to the COVID-19 pandemic, and it was a period of active communication through the university’s self-moderated discussion boards. To collect data for this study, we employed a self-selection approach where individuals who met specific criteria voluntarily participated. We encouraged all members of the university community, including students and faculty, to post promotional messages on the university’s self-moderated discussion boards.

The promotional messages were directly authored by the researchers and included information about the research’s purpose and significance, participation methods, criteria for selecting and excluding research participants, and a link to the survey (using Google Forms). Participants who believed they met the research criteria, such as being female university students in their 20s or having experienced menstrual cramps, were directed to the survey link and asked to confirm their eligibility. Those who met the selection criteria were provided with an ‘Informed Participant Information Sheet’ detailing the research’s purpose and participation procedures, and they were given the opportunity to provide their online consent. The survey commenced after this entire process was completed.

This self-selection approach was chosen due to the online nature of our study and the unique circumstances of the COVID-19 pandemic, which made traditional sampling methods less feasible. It has been demonstrated as an effective method for recruiting research participants in this context.

The promotional messages remained posted until all necessary participants for the research were recruited. Following the recruitment of all required participants, a message was posted expressing gratitude for research participation and announcing the conclusion of data collection. The data collection period for this study occurred from September 1st to September 19th, 2021.

### Research tools

#### PMS

The level of PMS was measured using the Shortened Premenstrual Assessment Form (SPAF) developed by Allen et al. [26] and translated by Lee et al. [27]. The SPAF consists of 10 items eliciting information on the level of each of the PMS symptoms that develop during the premenstrual week. Each item is rated on a 6-point Likert scale (1 = strongly disagree, 6 = strongly agree), with the total score ranging from 10 to 60 points, where a higher score indicates more severe PMS symptoms. The cut-off of 27 points was used to distinguish between PMDD and non-PMDD cases. The Cronbach’s α value was 0.91 in the study of Lee et al. [27] and 0.87 in this study.

#### Stress

The level of stress was measured using the Korean version of the Perceived Stress Scale (PSS) developed by Cohen et al. [28] and translated by Lee et al. [29]. This scale consists of 10 items, and each item is rated on a 4-point Likert scale (0 = almost never, 1 = sometimes, 2 = fairly often, 3 = very often). A higher total score indicates a higher level of perceived stress. Cronbach’s α reliability coefficient was 0.82 in the study of Lee et al. [29] and 0.79 in this study, indicating good internal consistency of the scale.

#### Depression

Depression was measured using the Korean version of the Center for Epidemiologic Studies Depression Scale-Revised (K-CESD-R) originally conceived by Eaton et al. [30] and translated into Korean and validity- and reliability-tested by Lee et al. [31]. This tool consists of 20 items eliciting information on participants’ weekly frequency of depression symptoms. Each item is rated on a 4-point Likert scale (0 = not at all or less than one day, 1 = 1–2 days, 2 = 3–4 days, 3 = 5–7 days or nearly every day for 2 weeks). The higher the total score, the higher the level of depression, with 0–20 points defined as normal, 21–40 points as risk, and 41–60 points as high-risk groups. Cronbach’s α reliability coefficient was 0.98 in the study of Lee et al. [31] and 0.91 in this study.

#### Sleep disturbances

Sleep disturbances were measured using the General Sleep Disturbance Scale (GSDS) developed by Lee [32] and translated by Choi et al. [33]. This tool consists of 21 items asking for the frequency of sleep problems in the past week grouped into six factors: sleep onset, maintenance of sleep, quality of sleep, quantity of sleep, fatigue and alertness at work, and use of substances to help induce sleep. The total score ranges between 0 and 147 points, where higher scores indicate greater likelihood of sleep problems. The lowest Cronbach’s α reliability coefficient was 0.75 in the GSDS developed by Lee [32] and 0.81 in this study.

#### Eating attitude problems

Eating attitude problems were measured using the Eating Attitudes Test-26 (EAT-26) developed by Garner et al. [34], with its Korean version standardized by Rhee et al. [35]. This tool comprises 26 items measuring diet-related problems clustered in four factors: self-control of eating and bulimic symptoms, preoccupation with being thinner, food preoccupation, and dieting. Each item is rated on a 4-point Likert scale (0 = never, 3 = always), with a higher score indicating higher likelihood of eating attitude problems. Based on a previous study [36], the total score range of 0–21 points is assessed as normal, 22–26 points as a possible eating disorder, and 27–78 as a severe disorder. Cronbach’s α reliability coefficient of the Korean version standardization study with women was 0.81; that of this study was 0.83.

### Data analysis

The data collected were analyzed using the SPSS version 26.0 program. The participants’ characteristics were analyzed and presented in terms of mean, standard deviation, frequency, and percentage. Differences between variables were tested using an independent t-test and one-way ANOVA, Scheffe’s test was performed as post-hoc testing, Pearson’s correlation coefficient was used to calculate the correlations between variables, and multiple regression analysis was performed to identify the factors influencing PMS.

### Ethical considerations

This study obtained approval from the Institutional Review Board (IRB No. 2021-07-053) of the data collection institution, ensuring that participants could voluntarily participate and provide informed consent. To prevent any incidental harm to the participants, collected data were encrypted and stored with restricted data access to prevent unauthorized access. It was clarified that the data would not be used for purposes other than the research.

Participants were informed in advance that they had the right to discontinue their participation or withhold information at any time if they wished. It was also explained that participants could access counseling services within the university if needed due to emotional experiences during their participation. The research results were described to be used as foundational data for the development of interventions to assist individuals experiencing PMS.

In appreciation of their participation, a token of gratitude was provided for the time spent by the participants.

## Results

### Differences in the level of PMS according to the general and menstruation-related characteristics

The participants’ mean age was 22.56 years. Most had a normal BMI (66.4%), followed by underweight (16.8%), overweight (9.1%), and obese (7.7%). The majority of participants did not engage in regular exercise (60.8%), did not smoke (93.7%), and consumed alcohol (55.2%). A high proportion skipped breakfast (78.3%) and were not on a weight loss diet (76.2%). The majority drank 1–2 cups of coffee per day (91.6%) and 3–4 glasses of water per day (35.0%). Menstrual characteristics were also recorded, with most having menarche at age 12–13 (40.6%), regular menstrual cycles (65.0%), and 5–6 days of menstruation flow (59.4%). The level of dysmenorrhea was assessed using the Numeric Rating Scale, resulting in a mean pain level of 6.73 ± 2.16. Severe pain was reported by 66.4%, moderate pain by 23.8%, and mild pain by 9.8%. Painkillers were the most commonly used strategy for pain relief (72.7%). Significant differences in PMS were observed in BMI, amount of menstruation flow, and menstrual pain intensity. Post-hoc testing showed that PMS was significantly higher for those with severe pain intensity (7–10 points) compared to mild pain intensity (1–3 points) (Table 1).

**Table 1:** PMS according to general and menstruation-related characteristics (N = 143)

Variables	Categories	Mean ± SD	n (%)	PMS		
				Mean ± SD	t/F (p)	
Age (years)		22.56 ± 1.91				
BMI	Underweight^a)^		24 (16.8%)	30.54 ± 8.61	3.530 (0.017)	
	Normal		95 (66.4%)	36.32 ± 10.94		
	Overweight^b)^		13 (9.1%)	29.54 ± 8.65		
	Obese^c)^		11 (7.7%)	30.00 ± 14.85		
Regular exercise	Yes		56 (39.2%)	36.39 ± 11.35	1.886 (0.061)	
	No		87 (60.8%)	32.86 ± 10.66		
Smoking	Yes		9 (6.3%)	36.44 ± 12.49	0.617(0.538)	
	No		134 (93.7%)	34.10 ± 10.96		
Drinking	Yes		79 (55.2%)	34.16 ± 10.11	− 0.094 (0.925)	
	No		64 (44.8%)	34.34 ± 12.15		
Breakfast	Yes		31 (21.7%)	36.35 ± 13.12	1.056 (0.297)	
	No		112 (78.3%)	33.66 ± 10.37		
Weight loss diet	Yes		34 (23.8%)	35.71 ± 12.83	0.884(0.378)	
	No		109 (76.2%)	33.79 ± 10.43		
Daily caffeine intake (200 ml per cup)	1–2 cups		131 (91.6%)	34.19 ± 10.73	0.022 (0.978)	
	3–4 cups		9 (6.3%)	34.67 ± 13.05		
	≥ 5 cups		3 (2.1%)	35.33 ± 21.73		
Daily water intake (200 ml per glass)	1–2 glasses		31 (21.7%)	34.55 ± 11.06	1.013 (0.389)	
	3–4 glasses		50 (35.0%)	32.34 ± 10.53		
	5–6 glasses		36 (25.2%)	34.75 ± 10.95		
	7–8 glasses		28(18.2%)	36.85 ± 12.01		
Age at menarche	< 12 years		9 (6.3%)	33.67 ± 12.69	0.583 (0.627)	
	12–13 years		58 (40.6%)	33.98 ± 11.48		
	13–14 years		31 (21.7%)	32.55 ± 10.96		
	> 14 years		45 (31.5%)	35.97 ± 10.29		
Regularity of menstrual cycle	Regular		93 (65.0%)	34.29 ± 11.46	0.387 (0.680)	
	Irregular		46 (32.2%)	34.57 ± 10.11		
	Other		4 (2.8%)	29.50 ± 13.03		
Average menstrual cycle	≤ 25 days		11 (7.7%)	32.46 ± 9.56	0.408 (0.748)	
	26–29 days		59 (41.3%)	33.37 ± 11.94		
	30–33 days		43 (30.1%)	35.33 ± 10.53		
	≥ 34 days		30 (21.0%)	35.07 ± 10.64		
Average menstrual period	< 3 days		4 (2.8%)	29.75 ± 6.08	0.630 (0.597)	
	3–4 days		34 (23.8%)	33.62 ± 9.50		
	5–6 days		85 (59.4%)	34.09 ± 12.27		
	≥ 7 days		20 (14.0%)	36.75 ± 8.29		
Amount of Menstruation	Low^a)^		10 (7.0%)	40.30 ± 12.96	4.241 (0.016)	
	Normal		103 (72.0%)	32.65 ± 10.92		
	High^b)^		30 (21.0%)	37.70 ± 9.48		
Menstrual pain intensity (NRS)	1-3^a)^	6.73 ± 2.16	14 (9.8%)	28.00 ± 11.40	4.228 (0.016)	
	4–6		34 (23.8%)	32.06 ± 9.54	(a < b)	
	7-10^b)^		95 (66.4%)	35.95 ± 11.11		
Main strategy to deal with menstrual pain	Enduring or resting		26 (18.2%)	32.46 ± 13.17	0.576 (0.632)	
	Warm pack		9 (6.3%)	37.00 ± 10.20		
	Painkiller		104 (72.7%)	34.59 ± 10.38		
	Other		4 (2.8%)	30.75 ± 16.38		

The measurement and analysis of the variables for the participants’ characteristics resulted in the following findings: Significant differences in PMS were observed in BMI (F = 3.535, *p* = .017), amount of menstruation flow (F = 4.241, *p* = .016), and menstrual pain intensity (F = 4.228, *p* = .016). In the post-hoc testing, however, only the menstrual pain intensity of 7–10 points (severe) was found to have a significantly higher PMS level than 1–3 points (mild) (Table 1).

### Level of PMS, depression, stress, sleep disturbances, and eating attitude problems

The severity of PMS, measured on a scale of 10 to 60, had a mean score of 34.25(11.03). According to the scoring criteria of the PMS severity measurement tool, 104 participants (72.7%) scored 27 or higher, indicating a level comparable to PMDD. The stress level, measured on a scale of 0 to 40, had a mean score of 17.48(5.22). The depression level, measured on a scale of 0 to 60, had a mean score of 15.55(11.87), with 72.7% of the participants within the normal range. The average score for sleep disturbance was 54.94(13.80), and 91.6% of the participants showed normal eating attitude (Table 2).

**Table 2:** Level of PMS, stress, depression, sleep disturbance, and eating attitude (N = 143)

Variables		n(%)	Range of Score	Mean ± SD
PMS	10 ~ 26 (non-PMDD)	39(27.3%)	10–60	34.25 ± 11.03
	27 ~ 60 (PMDD)	104(72.7%)		
Stress			0–40	17.48 ± 5.22
Depression	Normal (1–20)	104(72.7%)	0–60	15.55 ± 11.87
	Probable depression (21–40)	34(23.8%)		
	Definite depression (41–60)	5(3.5%)		
Sleep disturbances			0-147	54.94 ± 13.80
Eating attitude problem	Normal (0–21)	131(91.6%)	0–78	8.80 ± 8.36
	Possible eating disorder (22–26)	6(4.2%)		
	Severe eating disorder (27–78)	6(4.2%)		

### Correlation between PMS, stress, depression, sleep disturbance, and eating attitude problems

PMS was positively correlated with depression (r = .284, *p* = .001), stress (r = .274, *p* = .001), sleep disturbances (r = .440, *p* < .001), and eating attitude problems (r = .266, *p* = .001) (Table 3).

**Table 3:** Correlations between PMS, stress, depression, sleep disturbances, and eating attitude problems(*N* = 143)

	PMS	Stress	Depression	Sleep disturbances	Eating attitude problems	
PMS	1					
Stress	0.274 (0.001)	1				
Depression	0.284 (0.001)	0.548 (< 0.001)	1			
Sleep disturbances	0.440 (< 0.001)	0.411 (< 0.001)	0.594 (< 0.001)	1		
Eating attitude problems	0.266 (0.001)	0.259 (0.002)	0.254 (0.002)	0.227 (0.006)	1	

### Influencing factors on PMS

Multiple regression analysis was performed by inputting the menstrual pain intensity, which showed post-hoc differences among the participants’ general characteristics and major variables (depression, stress, sleep disturbances, and eating attitude problems). In the analysis of multicollinearity between independent variables, the tolerance limit was greater than 0.1 (0.933–0.975) and the VIF was less than 10 (1.026–1.072), demonstrating that there was no problem of multicollinearity. As a results of multiple regression analysis, menstrual pain intensity (*β* = 0.204), sleep disturbances (*β* = 0.375), and eating attitude problems (*β* = 0.202) were found to have an effect on PMS. The regression model was significant (F = 16.553, *p* < .001) with an explanatory power of 24.7% (Table 4).

**Table 4:** Influencing factors on PMS(N = 143)

Dependent variable	Independent variable	B	S.E.	*β*	t (*p*)
PMS	Constant	12.279	3.406		3.605 (< 0.001)
	Pain	4.747	1.716	0.204	2.766 (0.006)
	Sleep disturbances	0.300	0.060	0.375	4.979 (< 0.001)
	Eating attitude problems	0.267	0.099	0.202	2.685 (0.008)

F(p) = 16.553(< 0.001), R = .513, R^2^ = 0.263, Adj.R^2^ = 0.247, D-W = 1.987

NRS ≤ 7 (Severe) → 1

## Discussion

In this study, we examined the levels of PMS, stress, depression, sleep disturbances, and eating attitude problems among female college students who experience dysmenorrhea and determined factors associated with PMS.

The mean pain intensity of dysmenorrhea experienced by the participants of this study was 6.73/10, and the majority (66.4%) of participants experienced severe pain of seven points or higher. In this study, menstrual pain intensity was identified as the primary factor affecting the level of PMS, which is consistent with findings of previous research [37] and aligns with the finding that menstrual pain is a significant pathway to PMS [38]. The PMS measurement tool includes two items expressing pain, which can explain the high correlation between the level of PMS and the menstrual pain intensity. Moreover, the fact that menstrual pain intensity was identified as the first determinant of the level of PMS—a complex syndrome combining physical, behavioral, and emotional symptoms—can be interpreted as meaning that various symptoms of PMS are closely related to pain. According to a previous study [39], Korean female college students perceive menstruation as a factor that weakens their physical and psychological health more markedly than their U.S. counterparts, which is the reason behind their more intense pain reports. Therefore, it is necessary to understand dysmenorrhea as more than a mere physical symptom (i.e., dysmenorrhea is psychological as well as physical), reconsider menstruators’ perceptions of menstruation, and seek out diverse factors related to PMS. Moreover, it is necessary to establish appropriate coping strategies for each of those factors, going beyond medication or palliative treatment.

All participants in this study experienced dysmenorrhea. Among the participants, 72.7% were found to have scores on the PMS severity measurement equivalent to PMDD, indicating more severe symptoms compared to the typical PMS. This finding is in contrast to previous research [40] where 85% of adolescents experience premenstrual syndrome, of which only 38% are classified as PMDD. An accurate clinical diagnosis of PMDD requires daily prospective symptom assessment [27]. Due to the substantial potential prevalence of PMDD among female college students experiencing dysmenorrhea, a more proactive interest in this issue is necessary. Healthcare providers should guide the subjects to observe unpleasant premenstrual symptoms daily during their menstrual period in order to understand the degree of changes in symptoms. They should also educate them about the importance of proactive management and the methods to do so, based on the severity of the symptoms.

In this study, stress and PMS levels were positively correlated, which is consistent with previous research [41]. However, stress was not confirmed as a determinant of PMS, unlike a previous study that identified stress as a determinant of PMS [42]. Changes in hormonal levels throughout the menstrual cycle may affect sensitivity to stress, intensifying PMS symptoms by responding more intensely to stressors, particularly in the premenstrual or luteal phase [43]. Moreover, because stress acts as a significant factor for irregular menstrual cycles, thereby affecting changes in menstrual function [44], it is important to include stress as a variable for PMS intervention. Intervention strategies are required to strengthen individuals’ coping ability and protective factors against stress through an in-depth understanding of premenstrual emotional characteristics and negative emotion control strategies by college students themselves.

Whereas depression was positively correlated with the level of PMS, it was not confirmed as a determinant of PMS, which is partially consistent with the findings of previous research [4]. Given that the level of PMS is a significant risk factor for depression [19] and that college students who experience PMS are more likely to experience depression compared to those who do not [19], it is necessary to assess the risk of depression [11]. PMS induces negative emotions due to hormonal changes and lasts for a short time, disappearing with the onset of menstruation. However, because symptoms appear repeatedly over a long time and are not uniform but accompanied by a wide variety of physical, emotional, and behavioral symptoms, healthcare professionals need to understand the correlation between college students’ PMS experiences and emotional problems such as depression. They should prepare multifaceted interventional strategies capable of countering emotional problems more efficiently through preliminary assessments of depression severity and positive communication among family members.

However, sleep disturbance showed a positive correlation with PMS and was identified as the second determinant of PMS. This finding is similar to those of previous studies [45, 46] that revealed correlations between PMS, sleep duration, and sleep quality [45, 46] and identified unsatisfactory sleep quality as a determinant of PMS in female college students [47]. Our results also support the findings in a previous study that women with PMS had a shorter sleep duration, needed a longer time to fall asleep, and had lower quality of sleep compared with women without PMS [48]. Sleep, a basic human need, is an essential factor for individuals to maintain good health and satisfaction, including physical, mental, social, and spiritual functions and overall quality of life [49]. Since the outbreak of COVID-19, infection control policies, such as online education and social distancing, have changed various aspects of psychological health (e.g., stress and depression) [50] as well as lifestyle (e.g., sleep problems) [51]. Changed lifestyle patterns, in particular, lowered sleep quality due to the use of digital media before bedtime [52]. Sleep will have to be considered an important variable in PMS intervention, and a quality sleep management strategy should focus more on sleep quality than on sleep duration.

Finally, more serious eating attitude problems were found to be associated with higher levels of PMS, and eating attitude problems were thus identified as the third determinant of PMS level. This finding is in line with that of a previous study in which eating attitude problems—measured with EAT-26, the same tool used in this study—were observed more frequently in adolescents with PMS than in those without it [53]. Specifically, adolescents with PMS scored higher in emotional and uncontrolled eating behaviors. Moreover, a higher level of neurotic bulimia is more closely associated with moderate-to-severe PMS than no or mild PMS, with the highest level manifesting in cases diagnosed with PMDD, suggesting that the extent of eating attitude problems may differ depending on the premenstrual emotional state [54]. Given that PMDD is defined as a more serious form of PMS, eating attitude problems may increase as PMS severity increases. Therefore, it is essential to meticulously examine the severity of PMS symptoms experienced by the participants and plan customized interventional strategies accordingly.

PMS and dysmenorrhea are important health issues that women experience, and it is very meaningful to approach and solve them from various angles. The findings from this research are expected to greatly aid in supporting women experiencing PMS. The results of this study can help women improve their understanding of their dysmenorrhea and PMS symptoms and improve their ability to manage emotional difficulties and stress. Since intervention strategies should be customized for each individual, various approaches and methods should be used to manage stress, relieve depression, improve sleep quality, and solve dietary problems that suit the various situations and needs of women. Healthcare professionals, schools, universities, and other institutions should actively utilize these research findings to support women experiencing PMS.

As limitations of this study, it may be noted that because the study analyzed data obtained through a cross-sectional survey conducted at one university, it cannot be extended to discussing the causal relationships between PMS and individual variables such as stress, depression, sleep disturbances, and eating attitude problems. Additionally, its results cannot be directly generalized to all female college students. Despite this limitation, this study is significant because it has successfully measured the level of PMS experienced by female college students who have dysmenorrhea and identified three determinants of PMS, including emotional factors (i.e., depression and stress) and lifestyle factors (i.e., sleep, diet, and exercise).

## Conclusions

In this study, we examined the levels of PMS, stress, depression, sleep disturbances, and eating attitude problems among female college students who experience dysmenorrhea and determined factors associated with PMS. Menstrual pain intensity, sleep disturbances, and eating attitude problems were found to have an effect on PMS. Because PMS exacerbates in the late luteal phase of the menstrual cycle and is accompanied by predictable and periodic psychological and physical symptoms that are resolved with the onset of menstruation. Therefore, individuals should have a good understanding of their own menstrual pain patterns and be able to set up suitable coping strategies, such as exercise, alternative therapy, and painkiller medication. Furthermore, to minimize the experience of PMS symptoms and negative effects, individual coping strategies for emotional well-being should be sought, and effective interventions applied to help correct problems accompanying sleep and eating attitudes. We propose that further study should be conducted to examine whether the severity of PMDD changes according to menstrual pain, the pattern and degree of its change, and the paths through which sleep quality and eating attitude problems affect PMS.

## Data Availability

The datasets used and/or analyzed during the current study available from the corresponding author on reasonable request.

## List of abbreviations

BMI: Body mass index
COVID-19: Novel coronavirus disease 2019
DSM-5: Diagnostic and Statistical Manual of Mental Disorders, Fifth Edition
EAT-26: Eating Attitudes Test-26
GSDS: General Sleep Disturbance Scale
K-CESD-R: Center for Epidemiologic Studies Depression Scale-Revised
NRS: Numeric Rating Scale
PMDD: Premenstrual dysphoric disorder
PMS: Premenstrual syndrome
PSS: Perceived Stress Scale
SPAF: Shortened Premenstrual Assessment Form
